# Effects of departmental green anaesthesia interventions on carbon dioxide equivalent emissions: a systematic review

**DOI:** 10.1016/j.bja.2025.03.038

**Published:** 2025-05-09

**Authors:** Sascha Hammer, Michael Eichlseder, Christoph Klivinyi, Kordula Lang-Illievich, Angelika Moser, Alexander Pichler, Nikolaus Schreiber, Philipp Zoidl, Helmar Bornemann-Cimenti

**Affiliations:** 1 Department of Anesthesiology and Intensive Care Medicine, Medical University of Graz, Graz, Austria; 2 Department of Anesthesiology and Intensive Care Medicine, LKH Güssing, Güssing, Austria

**Keywords:** carbon emissions, carbon dioxide equiavalent emissions, education, greenhouse gas, sustainability

## Abstract

**Background:**

Anaesthesia contributes to greenhouse gas emissions and can play a crucial role in reducing the carbon footprint of the global healthcare sector. The aim of this systematic review is to identify which departmental interventions influence estimated carbon dioxide (CO_2_) equivalent emissions of anaesthesia and to quantify their reductions.

**Methods:**

A systematic literature search was conducted through four major electronic databases (Cochrane Library, Embase, MEDLINE, and PubMed) for studies investigating the changes in CO_2_ equivalent emissions per anaesthetic before and after departmental green anaesthesia interventions. Data were extracted by two independent reviewers. The primary outcomes were mean decrease of CO_2_ equivalents in kilograms per anaesthetic and total decrease in CO_2_ equivalents in tonnes. The mean effect was calculated as percentage change per patient and in total.

**Results:**

Of 3987 screened studies, 13 met the criteria for quantitative synthesis and showed low to moderate risk of bias. The following types of departmental sustainability interventions were found: education of staff, decreased use of desflurane and sevoflurane, promotion of TIVA, use of low fresh gas flows, proper waste management, and formation of green teams. The postinterventional total mean decrease of CO_2_ equivalents in kilograms per anaesthetic was 68.2% (18.6%) and in total tonnes was 75.2% (16.3%).

**Conclusions:**

Our analysis demonstrates the substantial CO_2_ reduction potential inherent in sustainable anaesthesia programmes. Currently available literature supports staff education on avoidance of desflurane, reduction of volatile anaesthesia, lower fresh gas flow, increased utilisation of TIVA, and implementation of proper waste management protocols in operating rooms as potentially effective interventions.

**Editor's key points**

• Departmental and institutional interventions can potentially influence estimated CO_2_ equivalent emissions as a result of anaesthesia procedures.
• This systematic review analysed specific approaches and quantified their effects.
• There were substantial CO_2_ reductions inherent in sustainable anaesthesia programmes.
• Banning or reducing use of desflurane was the most effective measure to reduce CO_2_ emissions, which is the major contributor to CO_2_ equivalent emissions from anaesthesia.
• Other approaches include staff education, reduced use of volatile anaesthesia, use of lower fresh gas flows, increased utilisation of TIVA, and implementation of proper waste management protocols in operating rooms.
• A number of questions remain unsewered, and more studies are needed to clarify the most cost-effective interventions.

Climate change represents one of the most significant long-term health threats in the 21st century, emerging as a primary concern for clinicians and healthcare leaders.^1^ In the western hemisphere, the healthcare sector contributes 3–10% of the total global greenhouse gas emissions.^2^ If considered an independent entity, this would make the healthcare sector the fifth largest carbon emitter on a global scale.^3^ Anaesthesia contributes significantly to the environmental impact of healthcare, as it is a highly technical and resource-intensive discipline.^4^ Anaesthesia personnel make critically important decisions, selecting from different anaesthesia methods and medications with widely varying estimated CO_2_ equivalent (CO_2_e) emissions.^5^ In addressing the substantial contributions of anaesthesia to climate change, volatile anaesthetics have garnered significant attention. This focus is justified owing to their highly potent greenhouse gas properties. Volatile anaesthetics undergo minimal metabolism within the body and are subsequently released into the atmosphere largely unaltered (≥95%), experiencing minimal chemical modifications.^6^ Sevoflurane and desflurane, the most frequently used gases, persist in the atmosphere for 1.1 (sevoflurane) and 14 yr (desflurane).^7^

Several strategies to reduce the carbon footprint of anaesthesia have been published in recent years. These include the utilisation of low or minimal fresh gas flow (FGF), prohibition of desflurane and nitrous oxide (N_2_O) in operating theatres, education and training of the operating room (OR) staff, minimisation of waste and overuse of materials, reduction of energy consumption, and use of methods other than inhalation anaesthesia such as regional techniques or TIVA.^2^^,^^8^, ^9^, ^10^ Recently, there has been a focus on abandoning use of volatile anaesthetics in adult patients.^11^ Multiple analyses suggest that use of TIVA has a reduced carbon footprint compared with inhalation anaesthesia.^6^^,^^12^, ^13^, ^14^ However, evidence supporting the adoption of a volatile agent-free anaesthetic approach remains limited. Data from Gonzalez-Pizarro and colleagues^15^ indicate that 64% of anaesthesiologists use halogenated gases daily during general anaesthesia. Evaluation of the effectiveness of different interventions to reduce CO_2_ emissions is infrequent and mostly focused on other sectors.^16^ This systematic review aimed to identify which departmental interventions influence CO_2_ emissions during anaesthesia and to quantify their reductions.

## Methods

This systematic review was designed and performed according to PRISMA guidelines.^17^ A systematic literature search was conducted to identify studies comparing the difference in CO_2_e emissions before and after implementation of a departmental environmental sustainability initiative.

### Literature search

The search was conducted using four major electronic databases (Cochrane Library, Embase, PubMed, and MEDLINE) covering the period from January 1946 to December 2024. The search strategy comprised three categories of search terms: population (e.g. anaesthesia and other names used to describe it), environmental aspect (e.g. carbon footprint), and intervention (Table 1). A secondary search was conducted by manually screening the references cited in relevant articles.

**Table 1:** Overview of the literature search strategy.

Objective	Search terms
Population	‘anaesthesia’ or ‘anesthesia’ or ‘anaesthesiology’ or ‘anesthesiology’ or ‘operating theatre’ or ‘operation room’
Environmental aspect	(‘sustainable’ or ‘sustainability’ or ‘environment’ or ‘environmental’ or ‘carbon footprint∗’ or ‘carbon dioxide’ or ‘Carbon emission∗’ or ‘life cycle assessment∗’ or ‘life cycle analysis∗’ or ‘waste’ or ‘climate friendly’)
Intervention	‘management’ or ‘department’ or ‘education’ or ‘strategy’ or ‘change’
Study type	‘article’ or ‘conference paper’ or ‘editorial’ or ‘letter’ or ‘preprint’ (unpublished, non-peer reviewed) or ‘case reports’ or ‘clinical study’ or ‘clinical trial’, all or clinical trial or collected work or ‘consensus development conference’, nih or ‘controlled clinical trial’ or ‘guideline’ or ‘multicenter study’ or ‘observational study’ or ‘randomized controlled trial’

### Study selection

The following inclusion criteria were used: empirical, prospective, and retrospective studies; conducted in human patients undergoing anaesthesia; and comparing the difference in CO_2_e emissions in total or per anaesthetic before and after interventions. Secondary sources of data including abstracts, reviews, case reports, and congress proceedings were not considered for inclusion. Other exclusion criteria were articles published in languages other than English or German. The study protocol was published and is accessible from the Open Science Foundation Registries.^18^

Two investigators (SH and PZ) independently assessed article abstracts and full texts for eligibility and subsequently conducted data extraction. The primary outcome of interest during data extraction was reduction of CO_2_e per anaesthetic and total CO_2_e. For studies that generally met the inclusion criteria but lacked relevant data, the respective authors were contacted to request supplementary information.

The included studies were categorised by study design in a narrative synthesis, and when criteria were met for quantitative outcomes, descriptive statistics were used. Data collection and analysis were conducted using RevMan Web (The Cochrane Collaboration, London, UK) and Microsoft Excel (Microsot, Redmond, WA, USA).^19^

### Risk of bias assessment

The risk of bias in each included article was further assessed using Cochrane's updated risk-of-bias tool for non-randomised studies of interventions (ROBINS-I V2).^20^ Assessments of quality and risk of bias were carried out in parallel by two reviewers (SH and HBC).

### Outcomes

Greenhouse gas emissions were quantified by conversion to CO_2_e, a standardised unit that enables comparison of various gases to CO_2_ emissions based on their Global Warming Potential over a 100-yr period (GWP100). The studies included in this systematic review translated drug quantities into CO_2_e utilising pharmacy procurement records and established life cycle assessments. These assessments use life cycle inventory analysis to evaluate the carbon footprint of a product from its raw material acquisition to its disposal.^21^ All included studies had converted their outcomes into CO_2_e.

### Statistical analysis and data management

Total decrease is presented as mean and standard deviation (sd). To convert data from mean values with 95% confidence intervals (CIs) to a uniform format of mean (sd), the Cochrane calculator integrated in RevMan Web was used.^19^ When outcomes were reported as median (interquartile range), mean (sd) was estimated according to the method of Luo and colleagues^22^ and Wan and colleagues.^23^ Microsoft Excel was used to calculate the total mean percentage decrease in CO_2_e in kilograms per anaesthetic and total CO_2_e in tonnes.

## Results

The implemented search strategy yielded 3987 articles, of which 429 were excluded as duplicates during prescreening and 3442 were excluded by title and abstract screening. There were 116 articles deemed eligible for full-text screening; of these, 12 studies met the criteria for qualitative synthesis. One study was identified through full-text reference list review, and as it met the inclusion criteria, we decided to include it into our systematic review. Finally, 13 studies were included into quantitative synthesis and subsequently underwent data extraction and analysis.

The included studies collectively represented data from seven countries (Australia, France, Germany, Singapore, South Korea, Switzerland, and USA) and were published between 2019 and 2024. The number of anaesthetics performed per centre ranged from 2843 to 115 504, and they used general anaesthesia with desflurane, sevoflurane, N_2_O, or TIVA for their outcomes.^24^, ^25^, ^26^, ^27^, ^28^, ^29^, ^30^, ^31^, ^32^, ^33^, ^34^, ^35^, ^36^

**Fig 1:**
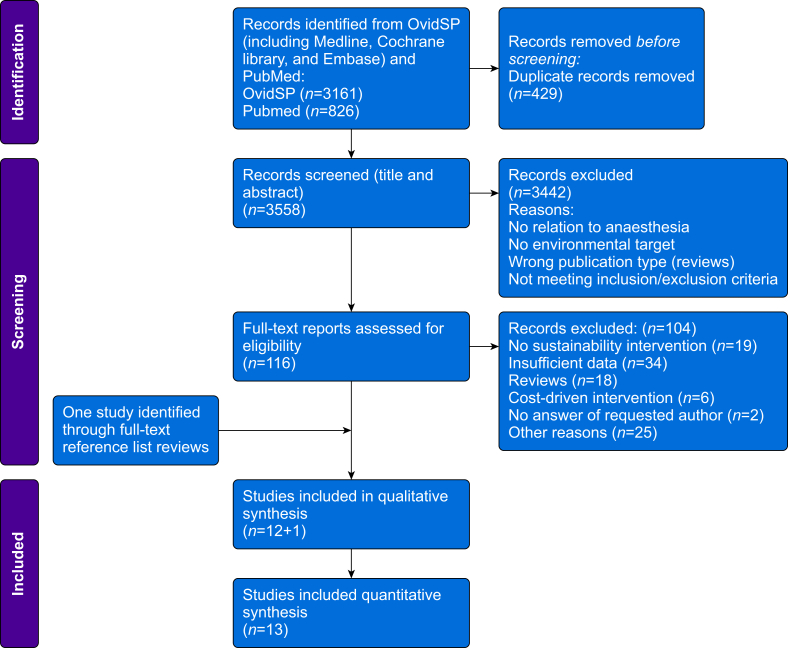
Study flowchart outlining the systematic literature search and screening process.

A PRISMA flowchart of the screening and selection process is presented in Fig. 1. The characteristics of the included studies are summarised in Table 2.

**Table 2:** Characteristics of included studies exploring their outcomes to reduce the carbon footprint of anaesthesia. CHF, congestive heart failure; CO_2_e, CO_2_ equivalent; FGF, fresh gas flow; N_2_O, nitrous oxide; OR, operating room; TIVA, total intravenous anaesthesia.

Author (year of publication)	Study country	Number of anaesthetics included	Time frame of intervention	Outcomes	Critical appraisal
Bernat (2024)^24^	France	97 422	2015–22	Desflurane and sevoflurane purchases decreased by 99% and 95%, respectively Propofol purchases increased by 11% Greenhouse gas emissions were reduced by 95%	No life-cycle assessments of propofol available Results being extracted from hospital pharmacy purchasing records
Chambrin (2023)^25^	France	115 504	January 2015 to February 2020	Median carbon footprint of desflurane and sevoflurane decreased by 84.3% Cost saving of ∼200 000 euros (£170 000, US$240 000) each year	Results were based on monthly pharmacy purchasing records Unable to assess the change in N_2_O use in Ors
Dölker (2024)^26^	Germany	No information	2018–22	44% reduction of wasted energy in ORs Reduction of purchased desflurane by 72%	Several unmeasured confounders (waste)
Gasciauskaite (2024)^27^	Switzerland	No information	Q2, 2021 to Q1, 2023	81% reduction in CO_2_e emissions 26% increase in the generation of plastic-related CO_2_e Economic impact decreased from 25 CHF (£22) per case to 21 CHF (£20) per case	Calculations of plastic consumption were only considered because of technical reasons Up to 26% propofol wastage because of excessive drawing
Glenski (2020)^28^	USA	No information	2019–20	Yearly carbon footprint of sevoflurane decreased by 75.0% Decrease of 20% in the number of sevoflurane bottles used per month 25% decrease in the average amount of sevoflurane used per anaesthetic	• Study at paediatric centre - no low-flows for inhalation inductions No transition to TIVA planned
Hafiani (2024)^29^	France	No information	2016–20	98% reduction of greenhouse gas emissions in 4 yr Annual cost saving of €36 690 (£30 820) Removal of the central piping for N_2_O led to annual savings of €4410 (£3700)	CO_2_ emissions factor was only measured for released gases, no propofol, waste, or materials were included
Hansen (2023)^30^	USA	No information	2017–22	87% reduction of CO_2_e Costs for volatile agents decreased over $140 000 (£115 000) annually from 2018 to 2021	• Emissions' data were missing from 468 of 61 052 (0.7%) anaesthesia records Used a GWP100 of 130 for sevoflurane rather than the 144 that is used in other studies
King Sin Ang (2023)^31^	Singapore	No information	March 2021 to April 2022	12 months reduction of 56.8% tonnes of CO_2_e Reduction of monthly desflurane use by 61.3% Cost savings of US$190 000 (£156 000) annually	Only changes of desflurane usage engaged
Park (2024)^32^	South Korea	2843	September 2022 to November 2022	CO_2_e per surgery performed under general anaesthesia decreased by 24.2% Total anaesthetic cost per surgery decreased from $14.6 (£12.0) to $12.4 (£10.2)	Study was conducted at two hospitals No follow-up, whether the effects of education continued over time Difficult to confirm N_2_O use during general anaesthesia using retrospective data analysis
Richter (2020)^33^	Germany	20 856	2020	Emissions from volatile anaesthetics decreased from 77% to 28.5% 67.4% reduction of CO_2_e per anaesthesia case Emissions from single-use disposable devices, packaging, and containers for fluids and drugs in 2017 (43.4 tonnes of CO_2_) and 2018 (41.8 tonnes of CO_2_)	• No complete lifecycle analysis for single use devices, packaging and containers Energy consumption of the OR was not included
Waberski (2023)^34^	USA	No information	Q3, 2019 to Q2, 2022	Purchase costs for volatile anaesthetics were reduced by 41% Decrease of 77.9% CO_2_e by reduction of isoflurane, sevoflurane, and desflurane Desflurane was eliminated from practice	• Generalisability to other institutions may be limited based on the resources and personnel available
Wyssusek (2022)^35^	Australia	No information	2016–21	Combined desflurane and sevoflurane emissions decreased by 87.88% Desflurane free in 2022 Annually costs of sevoflurane and desflurane combined decreased from $331 160.04 (£271 275.91) in 2016 to $137 991.72 (£113 038.48) in 2021, reduction of 58.33%	Calculations based on purchased bottles of volatile anaesthetics No collection of data regarding TIVA or regional anaesthesia Consumption of N_2_O was not assessed
Zuegge (2019)^36^	USA	60 736	2012–5	60.7% reduction of total CO_2_e emissions 64% reduction of CO_2_e emissions per case $25 046 (£20 517) cost savings per month	Pharmacy purchasing data for usage in the emissions calculations Increase in TIVA or regional anaesthetic rates not analysed

### Departmental interventions

As a basic intervention, all the included studies had staff education in common (Table 3).^24^, ^25^, ^26^, ^27^, ^28^, ^29^, ^30^, ^31^, ^32^, ^33^, ^34^, ^35^, ^36^ Ten studies urged anaesthetists to limit the use of desflurane.^24^^,^^25^^,^^27^^,^^29^, ^30^, ^31^, ^32^, ^33^, ^34^, ^35^ Study centres that have not yet discontinued the use of N_2_O have stated this as one of their departmental interventions.^27^^,^^29^^,^^30^ In addition to all anaesthesia-relevant factors, waste and energy reduction in the OR played a crucial role in three studies.^26^^,^^33^^,^^35^

**Table 3:** Different departmental interventions of the included studies. FGF, fresh gas flow; N_2_O, nitrous oxide; TIVA, total intravenous anaesthesia.

Authors	Staff education	Forming green anaesthesia teams	Limiting use of N_2_O	Limiting use of desflurane	Promotion of alternatives to halogenated gases (TIVA, regional anaesthesia)	Reducing FGF	Frequent updates and reminders	Waste and energy reduction
Bernat	X	X		X	X			
Chambrin	X	X		X				
Dölker	X							X
Gasciauskaite	X		X	X	X	X	X	
Glenski	X					X	X	
Hafiani	X		X	X		X	X	
Hansen	X	X	X	X		X		
King Sin Ang	X	X		X				
Park	X			X		X		
Richter	X			X		X		X
Waberski	X	X		X		X		
Wyssusek	X	X		X	X	X	X	X
Zuegge	X	X			X	X		

### Outcomes

All 13 studies reported a decrease in measured CO_2_e with the abovementioned interventions. Reduction of volatile anaesthetics, especially desflurane, was the most common outcome. The included studies varied between a 24% and a 98% decrease in CO_2_ emissions. The focus away from halogenated agents to TIVA in one study led to an 11% increase in propofol purchases.^24^ Gasciauskaite and colleagues^27^ reported an increase in plastics-related CO_2_e by 26% because of promotion of TIVA. The economic analysis in 11 studies found that the interventions led to cost savings ranging from €36 690 (£30 820) to €200 000 (£170 000) annually.^24^, ^25^, ^26^, ^27^^,^^29^, ^30^, ^31^, ^32^^,^^34^, ^35^, ^36^

**Fig 2:**
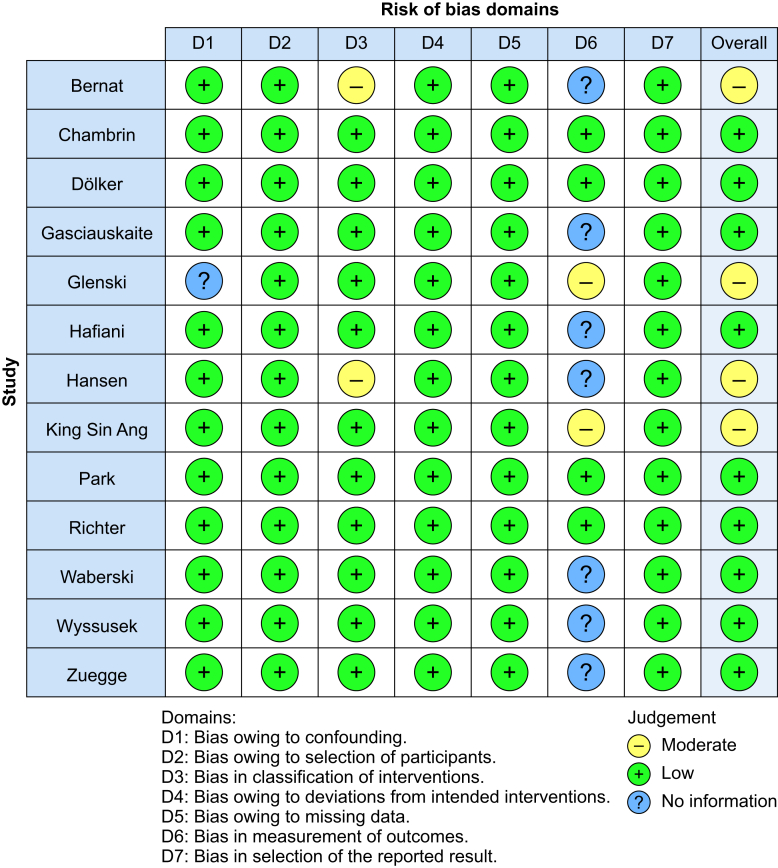
Risk of bias graph detailing the selection, performance, detection, attrition, reporting, and other biases among the 13 included studies. Green, low risk of bias; yellow, unclear risk of bias; red, high risk of bias.

Of the included studies, four had a moderate and nine had a low risk of bias (Fig. 2). The most frequent source of unclear risk of bias was related to the domain classification of interventions and the measurement of outcomes. Authors could not provide information of whether knowledge of the assigned intervention could have influenced the outcomes of their study.

All studies met the inclusion criteria for quantitative analysis. Both the primary outcomes, CO_2_e in kilograms per anaesthetic and total CO_2_e in tonnes, decreased in the post-intervention groups. The total mean decrease in CO_2_e in kilograms per anaesthetic was 68.2% (18.6%) and in total CO_2_e tonnes was 75.2% (16.3%) (Table 4).

**Table 4:** CO_2_ equivalents (CO_2_e) in kilogram per anaesthetic and total CO_2_e in tonnes of departmental sustainable interventions. Values are mean.

Author	Preintervention CO_2_e		Postintervention CO_2_e		% Reduction	
	Total (t)	Per anaesthetic (kg)	Total (t)	Per anaesthetic (kg)	Total	Per anaesthetic
Bernat	–	45.0	-	2.2	–	95.1
Chambrin	283.4	66.2	44,6	6.2	84.3	90.6
Dölker	58.0		25.5		44.0	–
Gasciauskaite	20.3	3.0	6.6	1.0	67.4	66.7
Glenski	114.0	–	28.5	–	75.0	–
Hafiani	911.0	–	18.0	–	98.0	–
Hansen	569.5	–	73.4	–	87.1	–
King Sin Ang	1477.7	–	637.7	–	56.8	–
Park	–	212.0	–	160.7	–	24.2
Richter	307.8	38.0	36.0	12.0	88.3	68.4
Waberski	172.0	–	38.0	–	77.9	–
Wyssusek	767.2	–	92.9	–	87.9	–
Zuegge	4724.1	163.0	1858.7	58.0	60.7	64.4
**Total mean decrease (****sd****)**	**855.0 (1357.3)**	**87.9 (81.3)**	**260.0 (560.2)**	**40.0 (62.9)**	**75.2 (16.3)**	**68.2 (25.2)**

## Discussion

From the 13 studies reviewed, it is clear that departmental environmental sustainability interventions can affect anaesthesia-related carbon emissions. On average, CO_2_ emissions were reduced by more than half. Looking at our results, the total mean decrease between studies varies. Ten studies reported a decrease of >60%; three were lower at 56.8%, 44.0%, and 24.2%.

### Educational interventions

Identifying ways to lower CO_2_ emissions is only one part of sustainability strategies. Another is how to implement them in anaesthesia departments and hospitals*.* Even though the studies differed significantly in terms of the interventions chosen, individual areas were identified that were considered by several authors to be the most important. For instance, all the included studies had staff education in common. It is essential to provide information frequently to all clinical staff involved (surgeons, nursing staff, anaesthesia). This information should cover the environmental impact of decision-making in patient care and different anaesthesia procedures.^37^ There were different approaches for sharing this information. Two studies invited speakers to hold presentations and give information to staff members.^31^^,^^32^ In five studies, training sessions were provided for all anaesthesia team members. Topics included use of sevoflurane over desflurane, reduction of FGF, monitoring of anaesthesia depth with the objective of using minimal doses, and TIVA theory.^24^^,^^27^, ^28^, ^29^^,^^34^ Others relied on journal clubs, posters in the OR, stickers on vaporizers, and e-mail reminders. It remains unclear which of these interventions was most beneficial, which will presumably depend heavily on the structure and size of the hospital.

### Inhalational anaesthesia

Attempts at decreasing the use of volatile anaesthetic agents can be variably effective. In two studies, use of N_2_O was completely discontinued with dramatic reductions in CO_2_e emissions.^29^^,^^30^ One study only achieved a 24.2% decrease in emissions, as many anaesthesiologists returned to using desflurane.^32^ One study observed pushback from staff on limiting access to desflurane, reportedly because of a sense of infringement on staff's clinical practice. This suggests that education needs to be frequent and include alternative options to encourage staff cooperation with practice changes.^31^ Furthermore, limiting access to an agent, such as removing desflurane vaporizers from the ORs, will further prevent backslide to old behaviour.

### Waste in the operating room

Anaesthesia is responsible for ∼25% of the waste generated during surgical procedures.^38^ To decrease the waste from anaesthesia, waste segmentation and limiting single-use devices are effective. In three studies, waste and energy reduction in the OR played a crucial role.^26^^,^^33^^,^^35^ Waste consumption decreased only slightly from 10.9 tonnes in 2017 to 10.5 tonnes in 2018 in one study.^33^ The authors stated that most plastic and paper waste was generated before a patient arrives, and in most cases the nursing staff is involved.^26^^,^^33^^,^^35^ Therefore, one of the most important points is the education of people involved in waste segregation, which in most cases is the nursing staff.^39^

### Formation of green teams

Creating green teams at the ground or higher level (department or hospital management) gives people the opportunity to discuss strategies, changes, and other ideas regarding sustainable health care.^40^ They should consider how to adapt available evidence to local circumstances. Seven of the studies formed green or sustainability teams in their department as interventions.^24^^,^^25^^,^^30^^,^^31^^,^^34^, ^35^, ^36^ Within green team meetings, members can discuss specific topics such as the emission profile of desflurane, sevoflurane, and other gases, overuse and waste of medications, and the effect of different FGF. Green teams were used to generate specific ideas and to advertise their interventions within the clinic to ensure that changes would be sustainable.^24^^,^^25^^,^^30^^,^^31^^,^^34^, ^35^, ^36^

### Reduction of fresh gas flow

Decreasing the greenhouse gas emissions from volatile anaesthetic agents can be mitigated through implementation of low-flow anaesthesia (FGF <1 L min^−1^.^28^ This approach results in reduced consumption of volatile gases, decreased costs, and minimised waste production.^41^, ^42^, ^43^ Nine studies included this topic in their educational efforts through various approaches.^27^, ^28^, ^29^, ^30^^,^^32^, ^33^, ^34^, ^35^, ^36^ Default machine FGF can be reduced from 8 to 2 L min^−1^ or from 8 to 4 L min^−1^.^30^^,^^34^ As a part of the technical solution, the machine alerted if volatile anaesthetics were detected, and the FGF was >2 L min^−1^ for >5 min.^30^ Another way to deal with high FGF rates is to use an automated control of circuit anaesthetic concentrations (ET control mode).^35^ Park and colleagues^32^ reported that there was no change in FGF before and after education, and raised concerns that compound A, which is produced when sevoflurane is used at low FGF, could cause renal injury.

### Technical approaches

The sustainability project of Hansen and colleagues^30^ monitored emissions of each anaesthesia provider in real time so that colleagues could compare the carbon footprint for their cases. The default anaesthesia workstation screen can display the cost per hour of selected volatile anaesthetics in real time. This immediate feedback serves as positive reinforcement, encouraging cost reduction by minimising unnecessary use of volatile anaesthetics caused by high FGF.^34^ Even if these technical solutions are not readily available, evaluating CO_2_ emissions and communicating success on a regular basis to staff members is a useful strategy. It generates a positive approach and increases motivation in the long term. Finally, mobilising feedback from healthcare staff and giving them space to discuss options should be included in departmental strategies.^30^

### Limitations

There are several potential limitations to our analysis. The primary limitation is the paucity of studies on this topic. It is anticipated that additional findings in this area will emerge in the coming years to enhance understanding of which measures are particularly efficacious. The heterogeneity of the study designs constrains conclusions and direct comparability of results. Hansen and colleagues^30^ did not include emissions from anaesthesia and surgery other than inhaled anaesthetic agents and used a GWP100 of 130 for sevoflurane, as opposed to the recently used value of 144. Furthermore, emission data of some cases (0.7%) were missing.^30^ Because of the retrospective nature of most studies, identification of causal relationships was not possible. Furthermore, there were no individual data available, so data were extracted from pharmacy purchasing records. This prohibits differentiation of patients treated with TIVA, volatile anaesthesia, or mixed i.v./volatile anaesthesia. To calculate plastic consumption, the cumulative number of infusion syringes and lines used throughout all anaesthesia procedures was considered, or was not observed owing to technical limitations. Another limitation is the absence of standardised propofol disposal procedures at the study centre, which did not allow inclusion of CO_2_e emissions from propofol disposal in their analysis.^27^ Finally, a meta-analysis of outcome data was not possible because of the heterogeneity of the included studies. Studies varied widely in design and population, so no statistical approach was deemed appropriate.

### Clinical perspective and open questions

Implementation of green anaesthesia requires collective effort rather than individual action. However, through effective leadership, team dedication, comprehensive educational programmes, and practice modifications, it is feasible to reduce CO_2_ emissions significantly. This systematic review demonstrates that a combination of interventions can have a substantial impact on reducing carbon emissions associated with anaesthesia practice when introduced as a departmental strategy. This includes avoidance of desflurane, promotion of physician education regarding greenhouse gas emissions, emphasis on TIVA and low FGF, and implementation of appropriate waste management systems in ORs. Current literature supports these findings, but their empirical foundation is limited. Consequently, there is a need for further systematic investigations to enhance our comprehension of the structural impediments to implementing institutional programmes and, most importantly, to identify solutions. On the basis of our review of the current literature, we paraphrased the following questions that remain unanswered and require further investigation. Firstly, what sustainable interventions have the greatest effects? What clinical environment (university hospitals, rural hospitals, or hospitals in countries with limited equipment and resources) benefits most from these changes? Secondly, what roles do advances in technology, green energy, staff mobility, and waste management play in green healthcare? Thirdly, from an economic perspective, what departmental changes are most cost-effective? Fourthly, how does regional anaesthesia fit into departmental sustainable strategies? Finally, how long does it take to obtain results after the strategies are implemented? Is it possible to gain a permanent shift towards sustainable healthcare or are there rebounds to be expected?

### Conclusions

Our analysis demonstrates the substantial CO_2_ reduction potential inherent in sustainable anaesthesia programmes. Banning or reducing use of desflurane is currently the most effective measure to reduce CO_2_e emissions, accounting for more than 60% of CO_2_e emissions from anaesthesia. The methods used in the available literature include staff education on avoidance of desflurane, reduced use of volatile anaesthesia, lower FGF, increased utilisation of TIVA, and implementation of proper waste management protocols in ORs.

## Authors’ contributions

Idea, hypothesis, and study design: all authors

Data acquisition: HBC, PZ, SH

Data acquisition and statistical analysis: HBC, ME, NS, SH

Data interpretation: all authors

Preparation of first draft of the manuscript: all authors

Commented on, edited, reviewed, and finally approved the final version of the manuscript: all authors

## Data availability statement

The data that support the findings of this study are available from the corresponding author upon reasonable request.

## Declaration of interest

The authors declare that they have no conflicts of interest.
